# The complex neurobiology of resilient functioning after childhood maltreatment

**DOI:** 10.1186/s12916-020-1490-7

**Published:** 2020-02-13

**Authors:** Konstantinos Ioannidis, Adrian Dahl Askelund, Rogier A. Kievit, Anne-Laura van Harmelen

**Affiliations:** 1 University of Cambridge, Department of Psychiatry, 18b Trumpington Rd, Cambridge, CB2 8AH UK; 2 Cambridgeshire and Peterborough NHS Foundation Trust/S3 Eating Disorder Service, Addenbrookes Hospital, Hills Rd Cambridge, CB2 0QQ, PO Box 175, Cambridge, UK; 3 MRC Cognition And Brain Sciences Unit, 15 Chaucer Road, University of Cambridge, Cambridge, UK

**Keywords:** Childhood maltreatment, Abuse, Neglect, Neurobiology, Resilience, Psychopathology, Genetics, Neuroendocrine, Inflammation, Brain structure, Brain function

## Abstract

**Background:**

Childhood maltreatment has been associated with significant impairment in social, emotional and behavioural functioning later in life. Nevertheless, some individuals who have experienced childhood maltreatment function better than expected given their circumstances.

**Main body:**

Here, we provide an integrated understanding of the complex, interrelated mechanisms that facilitate such individual resilient functioning after childhood maltreatment. We aim to show that resilient functioning is not facilitated by any single ‘resilience biomarker’. Rather, resilient functioning after childhood maltreatment is a product of complex processes and influences across multiple levels, ranging from ‘bottom-up’ polygenetic influences, to ‘top-down’ supportive social influences. We highlight the complex nature of resilient functioning and suggest how future studies could embrace a complexity theory approach and investigate multiple levels of biological organisation and their temporal dynamics in a longitudinal or prospective manner. This would involve using methods and tools that allow the characterisation of resilient functioning trajectories, attractor states and multidimensional/multilevel assessments of functioning. Such an approach necessitates large, longitudinal studies on the neurobiological mechanisms of resilient functioning after childhood maltreatment that cut across and integrate multiple levels of explanation (i.e. genetics, endocrine and immune systems, brain structure and function, cognition and environmental factors) and their temporal interconnections.

**Conclusion:**

We conclude that a turn towards complexity is likely to foster collaboration and integration across fields. It is a promising avenue which may guide future studies aimed to promote resilience in those who have experienced childhood maltreatment.

## Background

Up to a third of children growing up worldwide experience childhood maltreatment (CM) [1, 2], which can be defined as “*any act, or series of acts by a parent or caregiver that results in the (potential for) harm, or threat of harm, to a child*”. It comprises of abuse (i.e. sexual, physical and emotional) and/or neglect (i.e. physical and emotional) [3]. Children exposed to even a single episode of CM are at risk of repeated, more severe and more physical types of abuse or neglect [3–6]. CM is associated with poor functioning across a wide range of domains — it has been associated with problems directed towards the self (i.e. negative self-cognitions [7–9], alcohol abuse, impulse control problems [10] and suicidal behaviours [11]), interpersonal difficulties (i.e. increased peer rejection [12], social withdrawal [13], aggression and criminality [14]), physical health difficulties (i.e. failure to thrive, higher medical morbidity and mortality, e.g. see [3]), cognitive problems (i.e. impaired learning, working memory, verbal fluency and cognitive flexibility [15, 16]) and mental health disorders [13, 17, 18].

Although CM is associated with considerably lowered odds of good mental and physical health functioning later in life, a significant proportion of individuals with a history of CM function ‘better than expected’, when compared to other individuals exposed to CM. Those individuals, who may flourish in a single or multiple domains (e.g. socially, academically) [19], have been described as to be functioning ‘resiliently’ [20–22]. In this review, we highlight the complexity of neurobiological factors that aid such resilient functioning after CM by discussing the dynamic interplay of factors, which range from ‘bottom-up’ polygenetic influences to ‘top-down’ supportive social influences. In doing so, we argue that the neurobiology of resilient functioning after CM should be described and examined as a ‘complex dynamic system’. We suggest that future studies on resilient functioning after CM could move the field forward significantly by embracing a complexity theory approach. This would involve investigating multiple levels of biological organisation and their temporal dynamics in a longitudinal or prospective manner.

## Main body

### Resilient functioning after CM

Resilience denotes the ability of an organism to adapt to changing environments and cope with environmental challenges by shifting within its normal operating range [23]. There is considerable heterogeneity in the exact definitions used to describe resilience after CM (e.g. see [19, 24]). However, an emerging consensus in the field is that resilience refers to a positive outcome, or adaptation, following adversity [22, 25–27]. In individuals with CM, manifestations of this process are commonly inferred or determined in the aftermath of CM in the form of resilient functioning at a given time point from a given trajectory [22]. Considering the negative impact of CM on a broad range of domains, such resilient functioning after CM should be inferred from functioning across social, emotional, cognitive and/or behavioural domains [20].

**Fig. 1:**
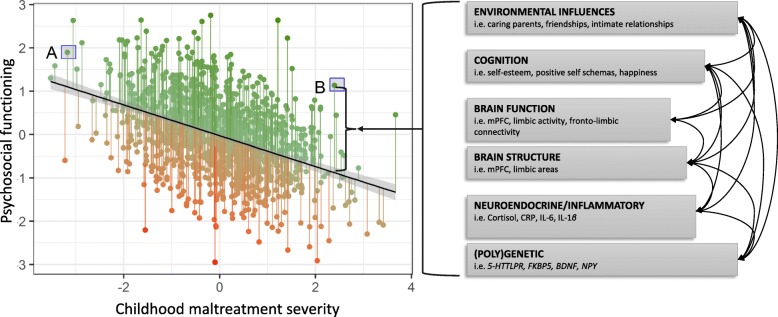
Resilient functioning and resilience factors. Individual resilient functioning as determined by the residual scores from the relationship between early life stress and psychosocial functioning. The residual scores reflect the extent to which an individual functioned better than expected (green lines, positive score) or worse than expected (red lines, negative score), given their history of childhood maltreatment (CM). Note that both axes represent factor scores with mean = 0 and SD = 1. **a** represents an individual who has experienced moderate CM and has lower resilient functioning scores than someone with lower psychosocial functioning who experienced severe CM (**b**)

Individual differences in the degree of resilient functioning should take into account the severity of the adverse experiences, such that resilient functioning refers to better mental wellbeing compared to others with a similar degree of adverse experiences [27]. In other words, a moderate level of functioning can indicate a higher degree of resilient functioning for someone with a severe history of CM when compared to someone with moderate or low CM. Figure 1 illustrates how multivariate techniques can be used to quantify resilient functioning as psychosocial functioning conditional on the degree of CM experiences. Here, the level of resilient functioning is inferred from the residuals of the relation between CM severity and psychological functioning across domains — the extent to which an individual is functioning better than expected given their CM experiences (implying resilient functioning, green lines) or worse than expected (implying vulnerable functioning, red lines) (Fig. 1, for a similar approach see [28–30]). Such a conceptualisation of resilient functioning entails an a priori strong association between psychosocial functioning and the measures of functioning (as the residuals will, by design, be highly correlated with psychosocial outcomes). However, it explicitly separates the two more clearly towards the extremes of CM severity — individual A, who has experienced little or no CM, will have lower resilient functioning scores than individual B, who experienced severe CM, even if the latter may have lower absolute psychosocial functioning (example highlighted in Fig. 1).

Resilient functioning after CM is thought to be facilitated by protective ‘resilience factors’ that help individuals to adapt and recover from, or compensate for, the sequelae of CM [21, 31]. These resilience factors comprise skills and resources linked to better outcomes in the face of adversity. Therefore, by measuring and/or assessing such resilience factors, an individual’s capacity for resilience could be assessed before stressor onset [32] — this is particularly important when considering interventions that could boost capacity for positive adaptations to adversity after CM. In the following paragraphs, we show how these resilience factors reside on multiple explanatory levels, ranging from genes to social influences [33], and describe how these factors are related to each other to facilitate resilient functioning after CM (Fig. 1). We refer readers to excellent narrative and systematic reviews of social, cognitive and behavioural [33], neurobiological [34–38], and psychobiological and molecular genetic factors of human resilience [23, 39] as well as animal models of resilience [36, 40].

### The complex interrelations of social, cognitive and neurobiological influences that facilitate resilient functioning after CM

**Fig. 2:**
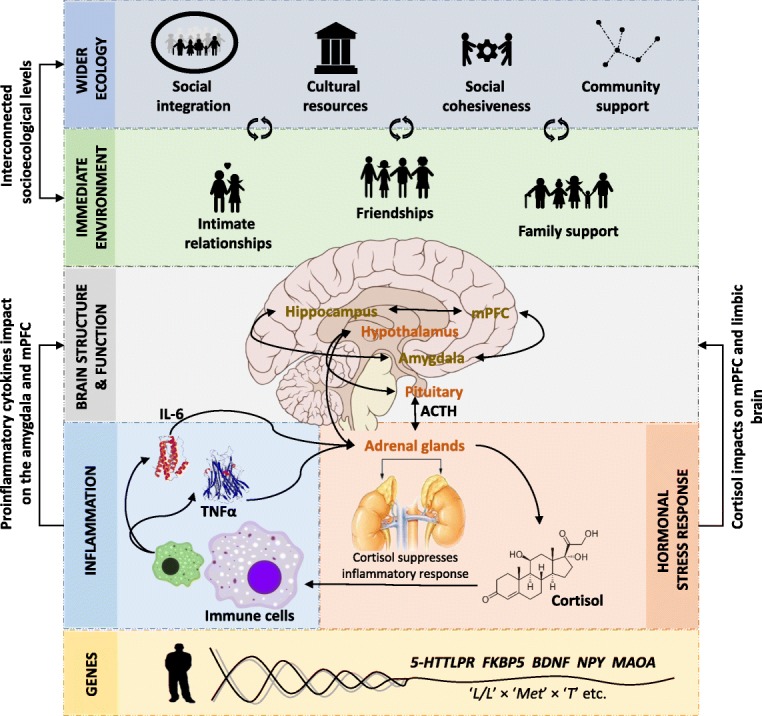
The complex neurobiology of resilience after childhood maltreatment (CM). Resilient functioning in those individuals who have experienced CM may be facilitated by larger prefrontal cortex (PFC) and hippocampal volume and connectivity, the ability to adequately regulate emotions and dampen stress responsivity, cortisol and proinflammatory baseline and responses, polygenic resilience effects, social support from the immediate environment, and the wider ecology. For readability, the location of the hippocampus is not correct. *5-HTTLPR* serotonin-transporter-linked polymorphic region, *ACTH* adrenocorticotropic hormone; *BDNF* brain-derived neurotrophic factor, *FKBP5* FK binding protein 5, *IL-6* interleukin 6, *MAOA* monoamine oxidase A, *mPFC* medial PFC, *NPY* neuropeptide-Y, TNFα, tumour necrosis factor-α

The human brain plays a key role in resilient functioning by orchestrating behavioural and physiological responses to stressors [41] (Fig. 2). The prefrontal cortex (PFC), for example, is critically involved in the executive control of cognitions, emotions and stress responses [42]. Surprisingly rudimentary properties of the PFC seem to be important for those brain functions, wherein larger PFC volumes are associated with improved performance in aspects of executive functioning (e.g. working memory) in healthy adults [43]. Two recent reviews of the neuroimaging literature suggest that resilient functioning in those with a history of CM (i.e. the absence of any mental health disorder as an outcome [44] or the absence of post-traumatic stress disorder [45]) has largely been examined only cross-sectionally and is related to altered volumes and/or function of (midline) PFC as well as to limbic regions and their functional connectivity [45–47]. For instance, in the multisite IMAGEN study (*n* = 1870 adolescents), larger right middle superior PFC volumes were shown to be associated with resilient functioning on multiple domains of functioning, including academic achievement, conduct, relationships and emotional health [48]. These studies provide some evidence that, cross-sectionally, larger PFC volume may be related to resilient functioning after CM. Further support for this idea comes from longitudinal behavioural studies revealing that smaller PFC volume after CM is linked to later poor cognitive functioning [49] and worsened illness courses [50]. However, it is not clear to what extent individual differences in the volume of the PFC are pre-existing vulnerability factors in those at risk or represent adaptive growth responses to stress in resilient individuals. To our knowledge, the only study that specifically examined PFC growth trajectories after CM found delayed maturation in the superior frontal gyrus in early adolescence, and that relative thickening of the superior frontal gyrus mediated the association between poor late adolescent functioning (i.e. decreased global functioning and lower rates of school completion) in boys who had experienced high maternal aggressive behaviour [51]. Thus, although some cross-sectional studies indicate that greater PFC volume is associated with resilient functioning after CM in adolescents, longitudinal evidence suggests more complex patterns. There is a clear need for further longitudinal research with designs that capture neurodevelopmental growth trajectories to examine the exact role of PFC volume and growth in resilient functioning after CM.

One likely explanation for the associations between PFC structure and resilient functioning is that the PFC plays a key role in the ability to regulate one’s emotions [52]. Such emotion regulation capacity is critically important in daily life and has been linked with a better ability to downregulate threat and stress responses as well as with improved mental health outcomes [53]. An increased emotion regulation capacity and associated brain functioning has been linked to resilient functioning after CM [54–56]. Such improved emotion regulation capacity may help resilient individuals to cope better with additional and/or daily life stress [57, 58] through their improved ability to downregulate and/or reappraise stress responses. The medial PFC plays an important role in inhibiting stress responsivity in the limbic regions [59, 60] and increased inhibitory activity in the rostral anterior cingulate cortex (ACC) has been linked to resilient functioning after CM [61]. This interpretation is supported by findings that healthy males with a history of CM showed limbic deactivation in response to stress [62] and that CM was negatively associated with amygdala and subgenual ACC responsivity to mild stress in healthy adults [63]. Such reduced activity of the limbic system is key, as limbic activity activates the hypothalamic–pituitary–adrenal (HPA) axis and stimulates the release of glucocorticoid hormones and proinflammatory biomarkers in the periphery [64] in two separate but inextricably intertwined biological systems — the HPA axis and the immune system [64]. In the next section we will describe how these processes have been linked to vulnerable and resilient functioning after CM.

#### The hypothalamic–pituitary–adrenal axis

The HPA axis is the core component of the neuroendocrine system that controls stress reactions, immune functioning and other physiological processes. In response to stress, the hypothalamus releases corticotropin-releasing hormone, which activates the release of adrenocorticotropic hormone from the anterior pituitary, which, in turn, stimulates the release of the stress hormone cortisol from the adrenal cortex. Cortisol is a glucocorticoid hormone that, among a wide array of functions, suppresses peripheral cellular and molecular inflammatory responses and binds with glucocorticoid receptors in the PFC and limbic structures to control brain development and responses [65].

In the context of CM, recurrent stress would lead to a chronically activated HPA system, which may lead to adrenal ‘fatigue’ and, via downregulation, to chronic adrenal stress hyporeactivity [66–69]. CM has diverse and profound effects on the endocrine system, as demonstrated in populations with a variety of adverse experiences, including single trauma [70], neglect [71] and social deprivation [72]. Results of these studies, summarised in recent meta-analyses and reviews, are mixed, with CM being related to both blunted and higher baseline cortisol, cortisol response to awakening and acute stress responses [73–76]. It has been suggested that the associations of cortisol with CM differ for patients with and without psychopathology [73]. Indeed, a recent meta-analysis that focused on healthy non-clinical populations reported that CM was associated with an increased cortisol awakening response and lower baseline cortisol levels [76]. However, to our knowledge, a direct comparison between clinical and healthy populations on baseline, awakening and stress responses for cortisol in individuals with CM has not yet been conducted.

The above evidence suggests that cortisol levels and responses may be related to resilient functioning after CM, although the specific direction of this relationship is unknown. In addition, glucocorticoids interact with other adrenal hormones such as the steroid androgen dehydroepiandrosterone (DHEA). DHEA acts as a natural antagonist to cortisol [77], may protect against the harmful effects of hypercortisolism [37, 78] and aids resilient functioning towards outcomes of depression [79, 80]. However, other findings suggest a more complex picture; for example, resilient functioning in a large sample (*n* = 677) of school-age maltreated children was positively associated with high morning cortisol, lower morning and afternoon DHEA, and higher morning and afternoon cortisol to DHEA ratios [78]. These findings emphasise the complex interplay of neuroendocrine factors that may facilitate resilient functioning after CM as well as the need for studies with large samples to yield precise and reliable estimates. A key pattern seems to be that maladaptive changes in the stress system after CM are associated with dysfunctional neurodevelopment, suggesting the presence of feedback loops operating on the interface of neuroendocrine and neural systems. For example, testosterone, when injected, can directly influence dominant or aggressive behaviour and is found to correlate positively with such behaviours [81], illustrating that the causal relationship may also be reversed — certain behaviours may themselves lead to an increase in testosterone, which in turn affects behaviour. Likewise, CM mediated the relationship between fractional anisotropy in corticomotor projections and baseline sympathetic nervous system activation, though not during cortisol administration challenge; these results may potentially suggest an altered neural circuitry having modulating effects in a network of neuroendocrine parameters of stress [82]. Furthermore, stress-sensitive hippocampal areas have been shown to be significantly smaller in children with CM, and CM moderated the positive linear relationship between left hippocampal volume and diurnal cortisol [83]. In sum, the processes that facilitate resilient functioning after CM may be reciprocal in nature, with simultaneous influences from neurophysiological properties to behaviour and vice versa.

Thus, while the neurodegenerative potential of glucocorticoids has robustly been demonstrated in preclinical studies [84, 85], the underlying mechanisms have not directly translated to human studies of CM. This suggests a more complex picture and a need to consider multiple biological levels (genetics, personality, behaviour, clinical phenotypes) to make sense of the interplay between neuroendocrine and neural factors [86]. One possible explanation for the difficulty in disentangling the causal effects between such processes is that they are not unidirectional, linear or additive, but rather highly dynamic and bidirectional, likely involving non-linear feedback loops between (sub)components of the systems [87]. This highlights the importance of future studies combining large samples with high temporal resolution of measurements as well as quantitative, complex systems approaches that are able to disentangle this web of reciprocal effects. Below, we highlight several cutting-edge tools that may offer researchers at least some traction on this highly complex and multifaceted problem.

#### The immune system

In response to stress, the sympathetic nervous system activates immune cells to propagate an inflammatory response. Specifically, via central and peripheral nervous system monoamine actions, the sympathetic nervous system propagates the release of proinflammatory biomarkers such as interleukin 6 and tumour necrosis factor-α (Fig. 2) [88]. Proinflammatory biomarkers play a key role in both stress reactivity and recovery [89–93]. Specifically, proinflammatory cytokines stimulate the HPA axis to release glucocorticoid hormones, such as cortisol, which in turn suppress the further release of cytokines from the immune system [94]. Over time, however, chronically elevated inflammatory responses lead to glucocorticoid resistance, with cortisol losing its anti-inflammatory efficiency [95]. Through this pathway, chronic stress in the context of CM may facilitate sustained inflammation in the periphery. Indeed, CM experiences have been linked to increased levels of peripheral inflammation biomarkers [96–99]. Changes in proinflammatory cytokines and glucocorticoid systems have also been associated with structural changes in brain regions crucial for emotion regulation and stress response [93, 100] (Fig. 2). Elevated proinflammatory biomarkers can cross the blood–brain barrier in various manners and negatively impact on the structure and function of brain regions involved in threat, reward and executive processing [89, 101]. Thus, the neural, immune and endocrine systems are closely linked in regulatory feedback loops that control stress responses and adaptation after CM.

Through their impact on the brain, proinflammatory biomarkers are thought to play a role in initiating and perpetuating mental health disorders [90, 102–109]. While low inflammation appears protective towards the development of mental disorders, there is currently no empirical evidence to support the notion that low levels of proinflammatory biomarkers facilitate resilient functioning after CM in humans. Some insights have been obtained by mechanistically robust animal studies, wherein stress-resilient mice had lower plasma corticosterone levels, lower PFC mRNA expression of corticotrophin-releasing factor and lower inflammatory circulating monocytes compared to stress-susceptible mice [110]; those mice also differed with respect to their hippocampal synaptic plasticity.

From the above, it is clear that HPA axis and immune interactions with the brain are involved in resilient functioning after CM. Future studies are needed to elucidate the exact role of the immune system in its interaction with HPA axis components as well as in relation to brain structure and function in resilient functioning after CM. Such studies may reveal empirical evidence supporting the role of immunological processing in resilient functioning after CM. Nevertheless, it seems likely that the mechanisms that connect neural, immune and endocrine systems to resilient functioning are closely linked, inherently dynamic and non-linear.

#### The role of polygenetics

Evidence from behavioural genetics suggests that individual differences in resilient mental health functioning has a significant heritable component, estimated at 50% [30]. Genes shape the neuroendocrine and immunological consequences of CM and therefore contribute to brain structure and functioning after CM. Indeed, a number of neuroimaging studies have identified gene × environment interactions [111, 112]. For example, brain-derived neurotrophic factor (BDNF Val66Met polymorphism) [113–116], serotonin-transporter-linked polymorphic region (5-HTTPLR) in SLC6A4 [50, 117], neuropeptide-Y (NPY) gene polymorphism rs16147 [118], monoamine oxidase A (MAOA) gene [119–121] or the FK506 binding protein 5 (FKBP5) gene [122] interact with CM to predict mental health outcomes. However, these findings must be viewed as preliminary because the field suffers from publication bias towards positive results [115, 123]. Indeed, a recent meta-analysis of 31 datasets containing 38,802 subjects found no support for a CM × *5-HTTLPR* interaction, although CM was found to have a main effect on risk for depression [124]. Moreover, in a recent overview of large population case–control studies of depression, no evidence was found for any polymorphism-by-environmental moderator effects, including CM [125].

Genetic effects are often polygenic [126, 127]. Thus, the presence or absence of certain haplotypes may interact with other genes (‘polygenic resilience factors’) to facilitate resilient functioning after CM. For example, the *BDNF met* allele was protective against the influence of the *5-HTTPLR S* allele risk on subgenual ACC and its structural connectivity with the amygdala [128]. However, establishing associations suggestive of ‘polygenic resilience factors’ is a daunting task — children bearing the haplotypes associated with positive outcomes later in life may also be growing up in more supportive home environments (and inherited both their haplotypes and a supportive home environment), whereas children with risk genes may be growing up in more adverse or ‘depressogenic’ environments [129].

Overall, there are significant challenges ahead for future research on the genetic determinants of resilient functioning after CM. Studies should use genetically sensitive designs because of potential intergenerational transmission of genes and environments that promote resilient functioning. They should also consider the complexity of polygenic influences in which a variety of haplotypes might interact with each other to promote resilient outcomes. Moreover, to ensure a more holistic, integrative understanding, such studies should ideally assess how polygenic and environmental influences interact with multiple levels of biological organisation simultaneously, rather than linking genetic markers directly with distal outcomes of psychopathology. Finally, to ensure the robustness and replicability of findings of effects that are likely to be small in size, large samples as well as other innovations, such as registered reports, are crucial [130].

#### The social environment

Positive environmental influences at all levels of the social environment (i.e. family, culture, social capital, social connectedness, community and their transactions) play a key role in promoting individual resilient functioning after CM [26, 131–134]. There is over 50 years of research showing the importance of social environmental influences on resilient functioning after CM [135]; whilst an appropriate inclusion of this literature would be warranted, this is outside the scope of the current review. As such, we refer readers to key papers on the importance of the social environment [26, 33, 131–138] and provide some examples below. Family support in adolescence as well as peer support is associated with reduced depressive symptoms and promotes resilient functioning across a range of domains in those who have experienced CM [12, 139]. The beneficial effects of social support may be mediated through neurobiological mechanisms that facilitate resilient functioning after CM; for example, experimental animal studies showing adverse effects of early life stress on neurobiology can be reduced through positive environmental changes during the animal equivalent of adolescence [140–143]. Specifically, environmental enrichment offered to juvenile rats who had been exposed to in utero stress increases their play behaviour, reduces emotionality, enhances anti-inflammatory cytokines [140] and reduces corticosterone response to immediate stress [142]. Similar findings have been reported in humans, where friendship interactions and higher social status were associated with a reduction in behavioural distress and distress-related medial PFC function alterations after exposure to simulated peer rejection in a lab setting [144–147]; in turn, this was associated with reduced peripheral inflammation (interleukin 6) levels [146]. Furthermore, earlier age of adoption or foster care from institutions has been associated with more typical amygdala discrimination between maternal and unfamiliar facial expressions [148] and more normative white matter development [149]. These studies provide preliminary empirical evidence that particular positive environmental factors (e.g. environmental enrichment, (new) familial support, social support, friendships) may support more resilient functioning through acting on core neurobiological processes (cytokines, HPA axis, brain structure and function), even after the maladaptive early environmental experiences occurred.

In addition to these immediate associations between social support and resilient functioning on an individual level, it is important to acknowledge the key role that immediate and wider social structures, such as communities, culture and societal integration, play in facilitating individual resilient functioning after CM. A socioecological system (e.g. a community of people with a shared cultural background) that is able to maintain the integrity of its supportive resources, infrastructure and social networks in the face of adversity (e.g. racism, colonisation, marginalisation, dispossession, societal disintegration, loss of language or culture) may form a crucial context for resilient functioning at the level of the individual [150–154]. For example, Panter-Brick et al. [155] showed that young Syrian refugees are able to function resiliently through drawing strength from positive relationships in their community. This is in line with previous findings and hypotheses that factors operating in the society of resettlement are critical for mental health outcomes among refugees [156]. For instance, cultural continuity in health services influenced positive mental health outcomes in the Aboriginal populations of Canada [154]. Furthermore, research in high-stress populations where little support is available (e.g. child soldiers and maltreated or racially marginalised children) has shown that individual-level characteristics account for less variability in outcomes compared to environmental characteristics (e.g. [157]; see [137]). Thus, the characteristics of the wider socioecological system are essential to understanding resilient functioning at the individual level.

While individual systems operate in constant interaction with multiple layers of ecology, resilience may stem from these complex interactions throughout development. This notion is sometimes referred to as ‘systemic resilience’ [138] and has been utilised to explore resilience with a focus on the family system (systemic resilience in families) [158] and/or the wider ecology (systemic resilience in multiple ecological layers) [138] as well as the interaction between systemic resilience and resilient functioning at an individual level in those who have experienced CM. Multisystemic resilience expands from the viewpoint of Developmental Systems Theory [159], in which a person’s development is affected by the complex interactions of several systems external to the individual, embedded in multiple ecological layers. Thus, responses to adversity in any one individual may be crucially affected by the family system, depending on the wider community and the prevailing values of their culture and society [150]. From the perspective of Developmental Systems Theory, contextual variables such as culture should be considered as an important moderator in studies on resilience. In fact, according to this perspective, the individual may not always be the most important locus of change in complex systems [137]. Therefore, future resilience research would benefit from consideration of the complex developmental interactions between multiple ecological systems to allow for the detection of important contextual mediators and moderators of systemic resilience.

### Towards a complex systems approach to resilience

Resilient functioning after CM relies on interactions that cut across multiple levels, ranging from the genetic to the societal level, that interact through regulatory loops to create a complex network of interactions (Fig. 2). As such, a more comprehensive understanding of resilient functioning after CM necessitates an appropriate conceptual framework to do so. We propose that complexity theory is one such framework, with its emphasis on complex systems as highly composite systems, built up from multiple interacting subunits [160], with bottom-up as well as top-down regulatory loops. If we consider resilient functioning as the higher-level manifestation of a complex developing system composed of subunits and regulatory loops, resilience factors can affect subunits or the nature of the interactions and regulatory networks. As such, resilience factors can be described as network nodes influencing interconnected and auto-connected ‘networks’ of symptoms (hybrid symptom-and-resilience networks) that dynamically guide clusters of symptoms through stress adaptation over time [161]. However, to truly help the field of resilience research move forward, complexity theory must offer analytical tools as well as a tractable conceptual framework to guide and inform research. Below, we briefly outline several promising quantitative approaches, innovations that are increasingly being applied in mental health research and are likely to confer considerable benefits on future studies of resilience.

#### The longitudinal dynamics of resilience

As outlined above, we conceptualise the neurobiology of individual resilience as an inherently dynamic process. This view is in accordance with Developmental Systems Theory, which proposes that resilience arises from complex dynamic interactions involving many processes within and between systems [32, 162]. Such systems comprise many kinds of interacting levels, ranging from microorganisms (e.g. the microbiome) to families, the economy and the global climate [32, 138, 162]. From the perspective of complexity theory, the temporal dynamics of complex systems can be described as deterministic, semideterministic and indeterministic, based on whether it is possible to predict past and future trajectories from their initial state [160]. By definition, the values taken by a complex system’s variables at any point in time (*T*_*x*_) describe the system’s state (*S*_*x*_), which can be represented by a point in a geometrical space [160]. The dimensions of such a system and space depend on the range of processes and variables included. Adding time, ‘space’ becomes ‘phase space’ — each point in the phase space represents a state in which the system could be at one time, corresponding to an assignment of particular values to the variables at a given instant [160]. The path that the systems follow through phase space can be described as the ‘trajectory of the system’.

**Fig. 3:**
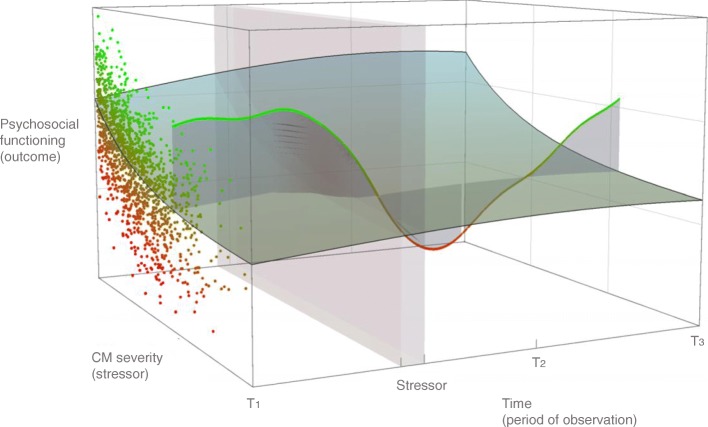
Trajectory of a complex resilience system in phase space. Resilience hyperplane plot of simulated data of childhood maltreatment (CM) severity (x-axis: stressor variable), psychosocial functioning (y-axis: outcome variable) and time (z-axis: period of observation), created by fitting a polynomial regression surface determined by numerical predictors of x, y and z using local fitting. An individual trajectory was hypothesised to demonstrate a complex system trajectory above and below the regression plane. Data points above the hyperplane (green) characterise ‘resilient functioning’, whereas all data points below the hyperplane (red) characterise non-resilient functioning at any time point (cross-sectionally)

Using such trajectories in phase space, we can quantify resilience over time by studying how system states evolve from the beginning to the end of the observational period. Using the method described in Fig. 1, resilience can thus be quantified through phase space as the integration of the system’s trajectory against the regression surface. To illustrate this, we have plotted a hypothetical trajectory of a complex system (say an individual) with their scores of psychosocial functioning (y-axis) and CM severity (x-axis) through time (z-axis), within a cohort of individuals (only presented as data points in T_1_) (Fig. 3). We have also plotted a regression surface (‘resilience hyperplane’); all data points above the hyperplane (green) characterise ‘resilient functioning’, whereas all data points below the hyperplane (red) characterise non-resilient functioning at any point in time (cross-sectionally).

Using Fig. 3 we can demonstrate why better understanding of resilience necessitates longitudinal data and techniques — if we consider an individual’s (complex system) trajectory through phase space, measuring this individual’s resilient functioning at T_1_, T_2_ or T_3_ would result in variable resilient functioning scores (positive at T_1_, negative at T_2_, positive again at T_3_). As such, if measured cross-sectionally, the individual would be characterised as ‘resilient’ at T_1_ and T_3_, and ‘vulnerable’ at T_2_. Although these states may accurately reflect the functioning of an individual at that moment, it is the variability and trajectory that yield a true understanding of the dynamics of the system as well as better quantification of resilient factors that support ‘upwards’ trends. In Fig. 3, the shaded grey area represents a hypothetical period of adverse experience(s). By studying such a trajectory longitudinally, additional adverse experiences, whereby an external stressor affects an individual’s psychosocial functioning, would further enable the untangling of the role of mental health predispositions and would thus allow for a more detailed investigation of residuals as markers of resilient functioning. This would then allow for the investigation of resilience mechanisms, the underlying processes by which resilience factors may facilitate resilient functioning in the aftermath of CM.

Such resilience mechanisms may manifest at different levels of abstraction, for instance, as moderating or mediating effects [163, 164]. Moderators directly affect the strength of the relationship between some form of adversity and an outcome, providing either a buffering or an amplifying effect. For instance, we observed that individuals who experienced more negative life events showed a stronger association between their positive memory specificity and negative self-cognitions [165]. In other words, individuals who had access to more specific positive memories displayed resilience against negative self-cognitions after negative life events. Mediators may provide specific, temporally ordered mechanisms through which (e.g. negative) events have distal effects. In the same paper, we found that individuals with greater positive memory specificity experienced fewer negative self-cognitions, which in turn led to fewer depressive symptoms [165]. In other work, we demonstrated that children who experienced greater childhood adversity showed greater depressive symptoms 3 years later, in part due to the mediating mechanism whereby greater CM negatively affected both friendships and family support in the intervening years [12]. Both findings of moderating and mediating mechanisms can allow researchers to quantify the capacity of an individual for resilient functioning, even in the absence of any negative events having occurred. This could ultimately be used to understand and guide interventions that could boost the capacity of (groups of) individuals to display resilient functioning when exposed to adversity.

In sum, quantification of an individual’s trajectory through phase space and the degree to which it can be predicted (determined) based on a number of known parameters (values) for their initial conditions will confer various scientific and translational benefits, including early warning markers, identification of resilience factors and quantification of temporal changes during development. Next, we will examine how to better understand the nature of these trajectories.

#### Understanding attractor states

A key concept from complexity theory relevant to resilience is the notion of an ‘attractor’ in complex systems; the attractor is a region in n-dimensional space towards which an agent in an environment has a tendency to move or return. Complex systems may display a particular behaviour of how they move through phase space — after an intervention or stress in the system, they may have a transient period during which they move in a specific direction through phase space, before returning back to their ‘normal’ behaviour. The phase space points corresponding to this ‘normal’ behaviour form the system’s ‘attractor’. Previous work provides many empirical examples of such attractor states. For example, following the loss of a spouse or child, individuals often retain or return to their pre-loss mental health levels [166]. This concept is crucial to understanding resilience. Resilience can be theorised as an attractor; after interventions or stressors, the resilient system has the tendency to return to a particular area of the phase space in which its functionality has returned back to ‘normal’. In the ‘resilience hyperplane’ paradigm (Fig. 3), the presence of a ‘resilience attractor’ would suggest the tendency of a system to return towards the higher values of the y-axis (psychosocial functioning), within a range of cumulative stress (x-axis), as time passes following a stressor. In other words, the presence of a resilience attractor would indicate that a system would tend to return to the area of phase space that has a specific range of values characterising normal functioning. In turn, resilience factors are those influences that may have the capacity to push an individual’s attractor state to a more well-adjusted region of this high-dimensional phase space. It is important to note that resilience, as an attractor state, does not imply that the resilient system is rigidly seeking to return to its exact adaptive functioning of the past or that the adaptations are ‘specific’ or ‘permanent’. Rather, attractor states describe areas of phase space in which return to normal function may be achieved through transformative change or reorganisation and in which the capacity to flexibly find new solutions to new problems is embedded in the resilient system.

#### Statistical techniques to investigate complexity in resilience research

The inherent complexity and dynamic nature of resilience after CM has been outlined in some detail above. However, to allow true scientific progress, we must harness techniques that can translate, capture and render tractable this complexity. Only by doing so can we translate the scientific study of complexity into quantitative models and make progress towards the ultimate goal of facilitating early detection, prevention and treatment. To achieve this goal, there has been an emerging appreciation for statistical techniques that can capture the phenomena of interest in ways that do justice to their inherent complexity. For instance, new work has shown how a range of quantitative techniques can capture non-linear dynamics (e.g. [167, 168]), early warning signals (e.g. [169]), bifurcations and attractor states (e.g. [170]), processes that are often discussed (usually in a qualitative sense) to describe developmental trajectories across explanatory levels. Beyond the academic literature, more accessible online resources, put together by world-renowned experts in complexity theory [171], provide a valuable starting point for researchers interested in translating ideas from complex systems into quantitative approaches. Below, we highlight a small number of quantitative approaches readily available and refer readers to specialised literature for in-depth discussions of these techniques.

Techniques such as Structural Equation Modelling (SEM) [172] can be profitably used to integrate notions of mediation, moderation and integration across multiple levels and timepoints. SEM is an overarching method that incorporates path analysis as well as latent variables, which may have advantages when studying resilience in large datasets with many variables. Path modelling is a more flexible extension of regression analysis [172] and is well suited to study complex resilience factors and processes as it can integrate data and hypothesised relations from multiple explanatory levels. For instance, using path modelling, we recently found that recalling specific positive memories was associated with reduced cognitive and physiological vulnerability to depression over two time points in adolescents exposed to childhood adversity [165]. Path analysis can also be used to test hypotheses of mediation and moderation, which may be of particular relevance in resilience studies investigating whether resilience factors and mechanisms moderate and/or mediate the relation between CM and mental wellbeing. Combining mediation and moderation using, for instance, conditional process analysis [164] can simultaneously address questions about the mechanisms behind resilience (mediation) and the conditions governing the strength of the linking mechanisms (moderation). In addition, SEM can be useful for integrating, or reducing, high-dimensional data. Beyond simple data reduction, latent techniques enable multidimensional conceptualisations of resilient functioning (i.e. across symptoms, cognitions and personality traits; see [139]). SEM is more flexible than regression-based techniques and offers robust handling of missing values, which is important in longitudinal studies [172]. SEM can be used to examine comprehensive integrative resilience models, for example, Kievit et al. utilised SEM to examine a ‘watershed’ model of the complex interrelations of brain structure, cognitive function and general intelligence [173].

Most importantly for resilience studies is arguably the quantification of change over time. Latent growth curve modelling [174] is a particularly versatile technique that allows researchers to quantify trajectories of resilient functioning, recovery or illness in longitudinal data. This technique allows for the elucidation and examination of resilient functioning trajectories over time [175] by reducing the impact of measurement error. Moreover, it allows for relatively simple inclusion of predictors of trajectories, the modelling of latent or manifest subgroups with distinct trajectories, and the demonstration of individual differences in trajectories.

Another important, and rapidly emerging quantitative framework is that of network analysis, a method that specifically examines the interrelations among variables. Network analysis has been used profitably in fields of psychopathology to conceptualise disorders as complex emerging phenomena [176]. More recent innovations in psychometric network theory [177] can bridge the gap between confirmatory models (where specific causal hypotheses are tested) and models that allow, in principle, for the full complexity of all interactions. In addition to modelling the direct interactions of symptoms (to help explain phenomena such as depression), network approaches can be utilised to examine complex network systems. For example, we recently utilised network analysis to examine the complex interrelations of resilience factors and their relations with mental health symptoms in adolescents reporting childhood adversity [178], addressing the complexity of resilience. Resilient functioning results from complex interactions between multiple bodily systems [179] and network analyses make it possible to examine interactions between different symptoms and neurobiology at an unprecedented level of detail [180]. In sum, recent statistical innovations have the potential to approach questions of resilience using frameworks that fully embrace the complexity inherent in resilience research.

## Discussion

We argue here that resilient functioning after CM is facilitated by complex interactions between neurobiological, genetic and social factors. Embracing a complexity perspective and associated statistical methods may aid future research on the neurobiology of resilient functioning after CM. Below, we will highlight three further aspects that such studies should consider.

First, resilience is inherently dynamic [27], such that the trajectories and predictors of resilient functioning may change over time [6, 27]. This is in line with the emerging literature on resilience from the perspective of Developmental Systems Theory that focuses on complex (dynamic and multilevel) person-oriented models and discusses maladaptive pathways of development and turning points in people’s lives [138, 159, 162, 181, 182]. The implications of this are noteworthy — individuals who we describe as to be functioning ‘resiliently’ at one point in time may not be characterised as such at another, and the environmental and neurobiological factors that predict such resilient functioning may be dependent on the timing of assessment. For instance, in childhood, amygdala hypervigilance may be an adaptive response to a highly stressful environment (for example, in the context of CM, rapid detection of whether a parent is in a bad mood may help the child to avoid a negative confrontation with that parent, leading to ‘resilient functioning’ in the short term). However, when the individual grows out of that particular social milieu, amygdala hyper-reactivity may form a vulnerability to mental health difficulties [183–185]. From this, it should be clear that the neurobiological elements of resilient functioning after CM cannot be understood unless they are studied in conjunction with their temporal (and social) dynamics [27, 186–188], quantified by appropriate analytic strategies.

Second, adaptive neurobiological responses after CM may depend on the type and timing of CM during development. This is in line with the Developmental Systems Theory principles of decentrality and complexity (focus on multiple systems, adaptations and solutions require complex interactions between systems) [138]. Single traumatic experiences and repeated trauma can be quite distinct with regard to the neurobiological sequelae, healing and recovery [189]; the importance of understanding and differentiating repetitive trauma from other types of trauma is also reflected in the recent inclusion of ‘Complex Post-Traumatic Stress Disorder’ as a separate diagnostic entity in the ICD-11 [190, 191]. Nevertheless, it is also important to appreciate the possibility that such disorders may not be possible to define aetiologically at a single level, but rather require considering the causal processes that interact across levels [192]. Threatening (sexual, physical abuse) versus depriving (neglect) experiences may impact on differential brain mechanisms [193]. Moreover, different brain regions have different windows of vulnerability during development (i.e. the life cycle model of stress [194]). Indeed, there is some evidence that the type and/or timing of CM were a stronger predictor of depression [195], cortisol [76, 78] and inflammation biomarkers [196] than the accumulation of CM occurrences. In support of this idea, the time of CM influences the type of clinical presentation in adolescence [197] and its neurobiological impact [185]. In sum, there may be distinctive neurobiological processes that promote resilient functioning depending on the type and timing of CM experiences as well as the timing of the resilient functioning assessment; these processes should be the subject of future research.

Third, the severity of CM matters not only for the quantification of differences in resilient functioning but also for the neurobiological mechanisms at play. Adversity exposure itself may also facilitate resilient functioning. For example, milder and more manageable levels of stress might have a ‘steeling’ effect on the individual [198], thus promoting resilient outcomes to future stress, a phenomenon described as stress inoculation [199]. Such steeling against depression was mechanistically demonstrated in mice using predictable mild chronic stress [200]. In contrast, high levels of stress have been associated with stress amplification/sensitisation or calibration effects [58, 201–203] (for extensive overviews see [204, 205]). This evidence demonstrates that a detailed understanding of resilient functioning after CM is contingent on a proper understanding of the nature and severity of CM experiences.

Finally, although a thorough discussion is beyond the scope of this manuscript, there are many intraindividual cognitive characteristics as well as interindividual family, school, social, and cultural influences that play a critical role in resilient functioning after CM [135–137, 158]. For instance, low ruminative tendencies, high autonomy, high self-esteem and self-efficacy affect resilient functioning after CM [33, 206, 207]. A recent systematic review of the literature suggests key roles for emotion regulation, cognitive skills, empathy and positive outlooks in resilient outcomes in children [136]. Indeed, positive views regarding the cognitive triad of self, the world and the future as well as the ability to remember specific positive events have been associated with a higher level of resilient functioning after CM [165, 208–210]. Moreover, self-reliance, self-confidence and interpersonal reserve promote resilient adaptations in children with a history of CM [211]. On an interindividual level, positive relationships with caregivers, friends, teachers or other adults, a safe and orderly school environment, student academic achievement, community cohesion and links with cultural identity, including spiritual beliefs, are related with resilient outcomes in children [136]. These findings are crucial, as they suggest, at least in principle, promising intervention targets to facilitate resilient functioning. Thus, neurobiological, genetic, cognitive and social factors play a key role in facilitating resilient functioning after CM and should be considered in future research.

## Conclusions

Resilient functioning after CM is governed by complex interactions between multiple biological and social levels. To further enhance our understanding of resilient functioning after CM, the field may benefit from embracing a complexity theory perspective involving the use of designs that allow the characterisation of resilient functioning trajectories, attractor states and multidimensional, multilevel assessment of functioning. This would include breaking free from reductionist conceptualisations suggesting that biological factor ‘X’ always ‘underpins resilience’ and acknowledging that resilience refers to the behaviour of a complex system that is high-dimensional and consists of dynamic interactions between multiple explanatory levels. Therefore, resilience should be studied using tools capable of capturing this inherent complexity. Such an approach involves the need for large, longitudinal studies on the neurobiological mechanisms of resilient functioning after CM that cut across and integrate multiple levels of explanation (i.e. genetics, endocrine and immune systems, brain structure and function, cognition and environmental factors) and their temporal interconnections. A turn towards complexity is likely to foster collaboration and integration across fields. It is a promising avenue towards guiding future studies aiming to promote resilient functioning in those who have experienced CM.

## Supplementary information

**Additional file 1: Figure S1.** Trajectory of a complex resilience system in phase space (mp4 version). See legend for Fig. 3 for detailed explanation

## Data Availability

Not applicable.
