# Correction to: The complex neurobiology of resilient functioning after childhood maltreatment

**DOI:** 10.1186/s12916-020-01657-z

**Published:** 2020-06-27

**Authors:** Konstantinos Ioannidis, Adrian Dahl Askelund, Rogier A. Kievit, Anne-Laura van Harmelen

**Affiliations:** 1 University of Cambridge, Department of Psychiatry, 18b Trumpington Rd, Cambridge, CB2 8AH UK; 2 Cambridgeshire and Peterborough NHS Foundation Trust/S3 Eating Disorder Service, Addenbrookes Hospital, Hills Rd. Cambridge, CB2 0QQ, PO Box 175, Cambridge, UK; 3 MRC Cognition and Brain Sciences Unit, 15 Chaucer Road, University of Cambridge, Cambridge, UK

**Correction to: BMC Med 18, 32 (2020)**

**https://doi.org/10.1186/s12916-020-1490-7**

The original article [1] mistakenly omits an acknowledgement to Raffael Kalisch who the authors would like to thank via this Correction article.

## References

[1] Ioannidis K (2020). The complex neurobiology of resilient functioning after childhood maltreatment. BMC Med.

